# Comparative miRNAome analysis revealed different miRNA expression profiles in bovine sera and exosomes

**DOI:** 10.1186/s12864-016-2962-1

**Published:** 2016-08-12

**Authors:** Ke Zhao, Guanxiang Liang, Xu Sun, Le Luo Guan

**Affiliations:** 1 Department of Agricultural, Food and Nutritional Sciences, University of Alberta, Edmonton, AB T6G2P5 Canada; 2 College of Medicine, Xi’an Jiaotong University, Xi’an, Shaanxi 710061 China

## Abstract

**Background:**

Extra-cellular components, such as serum and exosome, have drawn great attention as a readily accessible source of biomarkers for mammalian health. However, the contribution of different blood components to the signature of respective microRNAs (miRNAs) remains unknown, especially in cattle. In this study we aimed to investigate the miRNAs from bovine sera and exosomes, and to provide insights into their future applications.

**Results:**

Blood collected from four healthy dairy cows were used for this study. The serum and exosomal RNAs were extracted using two commonly used commercial kits (Norgen and Invitrogen total RNA isolation kit), respectively. The miRNA profiles were then generated using RNA-seq. Sera had higher complexity of miRNAome consisting of 328 ± 17 miRNAs, while less number of miRNAs (260 ± 15, *P* = 0.001) was detected in exosomes. The profile of total detected miRNAs in sera and exosomes was different, while exosomes represented about 78 % of total miRNAs expressed in sera, suggesting that exosomes are the major miRNAs carriers in bovine sera. A total of 24 and 3 miRNAs (RPM > 5) were exclusively expressed in sera and exosomes, respectively. In addition, 12 miRNAs were differentially expressed between sera and exosomes (FDR < 0.05), with the expression of four of them being further validated by stem-loop RT-qPCR. Moreover, functional analysis showed that uniquely and highly expressed miRNAs in sera were mainly related to diseases and disorders, while the predicted functions of those in exosomes were enriched in tissue development and lipid metabolism.

**Conclusion:**

Our results provide evidence that bovine sera and exosomes miRNAomes are different with regarding to the miRNA numbers, types and expressions. Based on their distinct profiles, miRNAs from sera and exosomes may reflect different aspects of physiological and pathological conditions in cattle. The functional analysis suggest that sera may be preferable for the purpose of detecting inflammation in cattle, while exosomes may be a better choice for monitoring the status of muscle development and lipid metabolism.

**Electronic supplementary material:**

The online version of this article (doi:10.1186/s12864-016-2962-1) contains supplementary material, which is available to authorized users.

## Background

As an important source of high quality protein for human consumption, cattle are considered to be one of the most important domestic animals. At present, intensive production systems have aggravated the health issues in cattle, such as mastitis [1], acidosis [2, 3], metritis [4], as well as welfare issues which have been leading to increasing foodborne and zoonotic risks to humans and economic losses for the industry. Due to the lack of validated early diagnosis procedures, most diseases and disorders are usually diagnosed at an advanced stage, and treatments for these pathologies are often expensive and ineffective. Therefore, there is an urgent need to identify reliable methods to diagnose the health status of cattle, which can then be used for early detection and monitoring disease progression.

Accumulating evidences suggest that expression patterns of microRNAs (miRNAs) in biofluids represent the *in vivo* status of many physiological changes and diseases [5–7], revealing that they can be served as diagnostic markers for multiple human diseases including cancers [8–11]. Blood is a non-invasive and the easiest obtained biofluid, and miRNAs in blood hold great promise to discover biomarkers for a wide range of diseases and biological processes [12–15]. Whole blood was the most frequently used biofluid in detecting miRNAs, but the outcomes could be biased due to the complexity of various cell types and components [16, 17]. Recently, the miRNAs in body fluids, such as plasma, serum, urine, saliva and sputum [18–20] have been used as informative biomarkers to assess and monitor the body’s physiological and pathological status due to their stability even under extreme conditions in human [21, 22]. In addition, it has been shown that the circulating miRNAs from plasma and serum could serve as potential biomarkers for livestock health and disease, such as miR- 26a for cattle early pregnancy [23], miR-19a and miR-19b for cattle heat stress [24], miR-29c and miR-375 for chicken puberty onset [25], and miR-122 for pig cardiogenic shock [26].

These free circulating miRNAs can be protected from nucleases by various types of carriers [27], such as exosomes. Exosomes are the most studied carriers, which are small (30–90 nm) and derived from the multivesicular body-sorting pathway [28–30]. Recent researches have proposed to use exosomal miRNAs for diagnositic markers in human diseases [31], since the miRNAs in exosomes have specific function and higher variability than blood cells [16]. In addition, the quantity of miRNAs in exosomes exhibited more difference between healthy individuals and cancer patients than that in sera [27]. However, the exosomal miRNA profiling needs extra steps in RNA extraction and which miRNAs (sera vs. exosomes) are more representative for physiological and health changes in cattle have not been defined.

The next-generation sequencing has made it possible to obtain highly detailed information of miRNAs on the types and abundance from various biomaterials [32]. However, consensus has not been reached with regard to the sample types used for isolation of total RNA (such as sera or exosomes), especially in cattle. To date, there is little information on miRNAomes in bovine sera and exosomes, which could be potential diagnostic biomarkers relating to cattle health. Therefore, the aim of the current study was to compare the bovine miRNAomes of sera and exosomes, and to provide insights into their future applications.

## Results

### MiRNA profiles of sera and exosomes

**Fig. 1:**
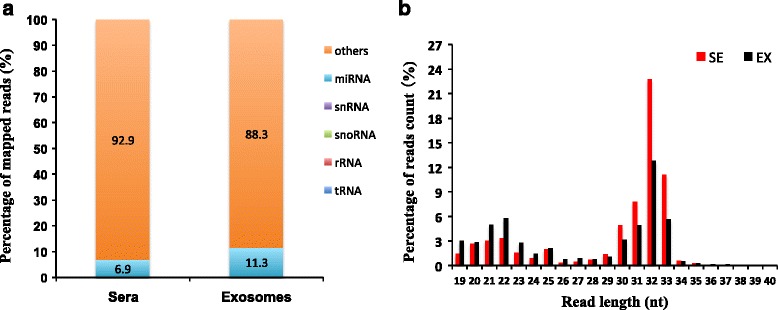
Profiling of small RNAs in cattle sera and exosomes samples. **a** The relative abundance of different classes of small RNAs. **b** Size and frequency distribution of detected small RNAs (19-40 nt). *SE* Sera, *EX* Exosomes

An average of 9.25 ± 1.44 and 15.70 ± 12.43 ng small RNAs were obtained from sera and exosomes (Table 1), respectively. RNA sequencing resulted in 29,692,695 reads for sera and 6,581,761 reads for exosomes, respectively. After quality and length filter, 22,745,381 reads (76.6 %) in sera and 3,960,646 reads (60.2 %) in exosomes were used for further analysis (Additional file 1: Table S1). In exosomes, higher proportion of reads failed the size trimming compared to sera (39.9 % vs. 23.8 %). After mapping to bovine genome (Baylor Btau 4.6.1), the proportion of annotated miRNAs was 6.9 % in sera, and 11.3 % in exosomes (Fig. 1a). And the reads mapped to tRNA, rRNA snoRNA and snRNA were low (< 1 %) (Fig. 1a) from both sera and exosomes with large proportion reads unidentified. Size distribution of the reads between 19 and 40 nt revealed 2 peaks at 19–25 nt and 30–33 nt for both sample types (Fig. 1b).

**Table 1:** RNA extraction from bovine sera and exosomes

Item	Kits	Serum (μL)	Elution (μL)	Total small RNA ± SD (ng)
Sera	Total RNA Purification Kit (Norgen, Thorold, CA)	200	50	9.25 ± 1.44
Exosomes	Total RNA Isolation Reagent and Total Exosome RNA and Protein Isolation Kit (Invitrogen, Carlsbad, USA)	800	50	15.70 ± 12.43

### Complexity and specificity of sera and exosomes miRNAomes

**Fig. 2:**
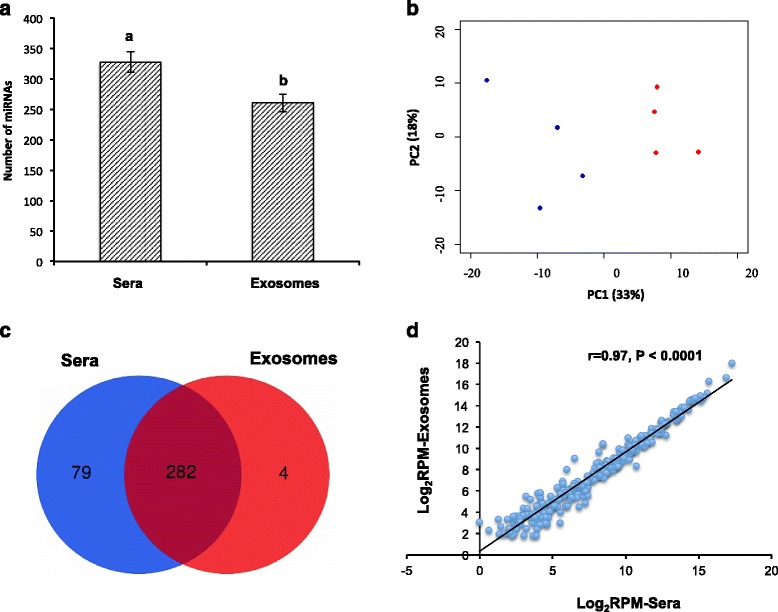
Complexity of miRNAs detected in the sera and exosomes. **a** Average number of miRNAs expressed in sera and exosomes, different letters (a, b) represent significant difference (*P* < 0.05). **b** Principle component analysis of the total detected miRNAs in sera and exosomes (blue dots represent sera, red dots represent exosomes). **c** Venn diagram showing the profile of miRNAs expressed in sera and exosomes. **d** Correlation analysis of the commonly expressed miRNAs in sera and exosomes

The miRNAs that were detected in at least two cattle with more than 1 reads per million total mapped reads (RPM) in sera or exosomes were considered as expressed miRNAs. Sera had higher number of miRNAs (328 ± 17) expressed, while significantly less miRNAs (260 ± 15, *P* = 0.001) were expressed in exosomes (Fig. 2a). The respective miRNAomes of sera and exosomes are available in Additional file 2: Table S2. Using principle component analysis (PCA) it showed that the miRNAs profiles between sera and exosomes were different (Fig. 2b). More uniquely expressed miRNAs were found in sera than those in exosomes, with 24 and 3 uniquely expressed miRNAs detected (with mean RPM > 5) in sera and exosomes, respectively (Table 2).

**Table 2:** Unique miRNAs expressed in bovine sera and exosomes

Sera		Exosomes	
miRNA ID	Mean RPM ± SD	miRNA ID	Mean RPM ± SD
bta-miR-493	16.39 ± 12.03	bta-miR-2284k	8.15 ± 6.87
bta-miR-196b	16.25 ± 5.54	bta-miR-6533	7.29 ± 8.56
bta-miR-1271	15.74 ± 4.79	bta-miR-9-5p	7.29 ± 8.56
bta-miR-708	13.41 ± 6.50		
bta-miR-628	13.09 ± 3.25		
bta-miR-18b	13.02 ± 7.63		
bta-miR-2285b	12.26 ± 3.69		
bta-miR-187	11.03 ± 15.55		
bta-miR-7861	10.96 ± 8.15		
bta-miR-95	9.88 ± 6.55		
bta-miR-543	9.61 ± 6.82		
bta-miR-424-3p	7.08 ± 6.55		
bta-miR-2299-3p	7.02 ± 8.54		
bta-miR-2448-3p	7.01 ± 3.55		
bta-miR-592	6.91 ± 5.45		
bta-miR-369-5p	6.44 ± 6.96		
bta-miR-2447	6.42 ± 8.91		
bta-miR-29d-5p	5.89 ± 5.02		
bta-miR-545-3p	5.55 ± 4.92		
bta-miR-9-3p	5.29 ± 6.72		
bta-miR-376b	5.18 ± 4.15		
bta-miR-495	5.13 ± 5.23		
bta-miR-183	5.08 ± 5.94		
bta-miR-196a	5.08 ± 5.94		

The expression is presented as  reads per million (RPM) with Mean RPM > 5. *SD* Standard deviation

**Fig. 3:**
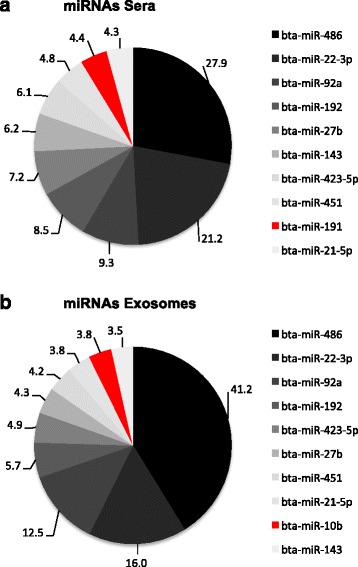
The pie chart of ten most abundant miRNAs. **a** Sera. **b** Exosomes. MiRNAs differed in two samples were colored in red

It was observed that 282 miRNAs were commonly expressed in sera and exosomes (Fig. 2c), and their expression profiles had a high correlation of 0.97 (Fig. 2d). From the commonly expressed miRNAs, the proportion of the top 10 miRNAs accounted 95.6 and 96.2 % of total reads for sera and exosomes, respectively, with the same predominant miRNAs identified. The comparison of the relative abundance of top 10 annotated miRNAs showed that sera and exosomes had similar profiles of abundant miRNAs except miR-191 in sera and miR-10b in exosmes (Fig. 3a, b).

In addition, 12 miRNAs were differentially expressed (DE) between sera and exosomes. The expression of miR-361, miR-16a, miR-146b and miR-24-3p were higher in sera, while the expression of other eight miRNAs (miR-328, miR-1249, miR-1306, miR-339b, miR-339a, miR-296-5p, miR-21-3p and miR-92b) were higher in exosomes (FDR < 0.05, Table 3).

**Table 3:** Differentially expressed miRNAs in sera and exosomes

miRNA ID	Log_2_ FC	Log_2_ RPM	*P* Value	FDR
bta-miR-361	1.03	9.72	3.01E-05	1.10E-03
bta-miR-16a	1.19	12.13	3.00E-04	7.50E-03
bta-miR-146b	1.89	6.69	3.00E-04	6.30E-03
bta-miR-24-3p	2.06	9.91	1.62E-10	1.08E-08
bta-miR-328	−2.93	8.40	5.74E-14	5.07E-12
bta-miR-1249	−2.74	4.97	2.00E-04	6.20E-03
bta-miR-1306	−2.44	7.44	1.44E-06	6.36E-05
bta-miR-339b	−2.29	9.87	3.19E-19	5.29E-17
bta-miR-339a	−2.29	9.87	3.99E-19	5.29E-17
bta-miR-296-5p	−2.09	5.40	2.00E-04	5.60E-03
bta-miR-21-3p	−1.76	9.12	7.43E-09	3.94E-07
bta-miR-92b	−1.16	10.74	2.00E-04	6.20E-03

### Experimental validation of miRNA expression

**Fig. 4:**
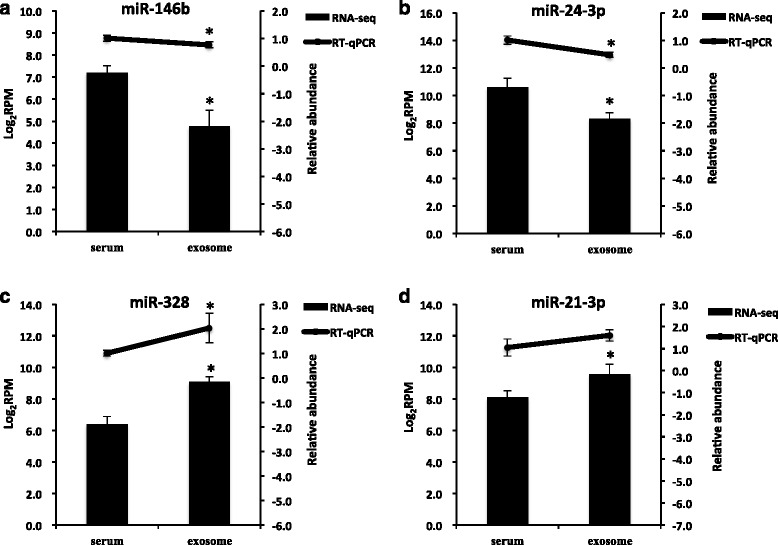
Expression of selected DE miRNAs between sera and exosomes detected by RT-qPCR and miRNA-seq. **a** Expression of miR-146b; **b** Expression of miR-24-3p; **c** Expression of miR-328; **d** Expression of miR-21-3p. miRNA expression from RT-qPCR represented by lines on the top and values are shown on the right vertical axis as relative abundance. miRNA expression from miRNA-seq represented by bars on the bottom and values are shown on the left vertical axis as log_2_RPM (normalized reads number). * on the top of lines or bars indicate significant (*P* < 0.05 or FDR < 0.05) difference between sera and exosomes. Data are presented as Mean ± Standard deviation

A total of 10 uniquely expressed and DE miRNAs that identified from miRNA-seq (four uniquely expressed miRNAs in sera: miR-196b, miR-196a, miR-18b, and miR-9-3p; two uniquely expressed miRNAs in exosomes: miR-2284k and miR-9-5p; four DE miRNAs between sera and exosomes: miR-146b, miR-24-3p, miR-328, and miR-21-3p) were selected for validation using stem-loop RT-qPCR. Three out of four DE miRNAs showed similar trend as detected by miRNA-seq. Specifically, the expressions of miR-146b and miR-24-3p were highly expressed in the sera, while miR-328 was highly expressed in the exosomes by miRNA-seq and RT-qPCR (*P* < 0.05) (Fig. 4 a-c). Although the expression of miR-21-3p, detected by RT-qPCR showed no significant difference between sera and exosomes (*P* < 0.1), its expression was numerically higher in exosomes, which was similar with the results detected by miRNA-seq (Fig. 4d). However, the expression of those sera or exosomes uniquely expressed miRNAs could not be detected by RT-qPCR (CT value < 35), which may be due to their low abundance and the relative low content of miRNAs in serum.

### Stability of the bovine sera and exosomes miRNAomes in healthy animals

**Fig. 5:**
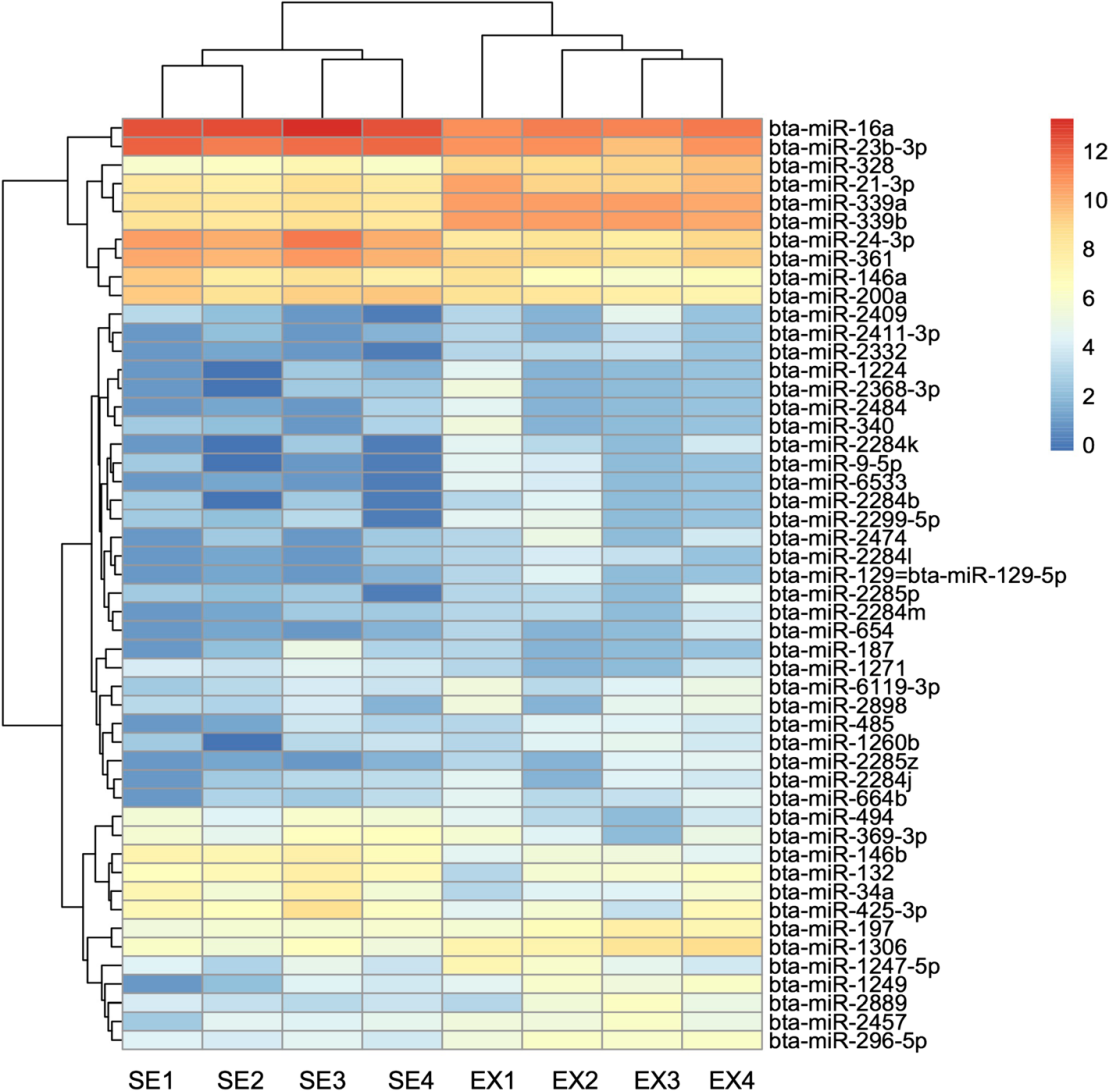
Heatmap generated by clustering of the 50 most variable miRNAs in sera (SE) and exosomes (EX) from four cattle. Colors represent different normalized sequencing reads number as indicated by the color bar (Log_2_RPM)

It is important to determine how miRNAomes varies among individuals, especially for the healthy animals. As the sera and exosomes were taken from the same four cattle, the individual variation between sera and exosomes reflected the stability of miRNAs in two types of samples. The high correlation was observed with *r* = 0.96 for sera (Additional file 3: Table S3) and *r* = 0.95 for exosomal miRNAomes (Additional file 4: Table S4) among four animals. The hierarchical clustering analysis on the 50 most variable miRNAs revealed that sera and exosomes were clustered to different groups (Fig. 5).

### Functional categories and pathways of uniquely expressed and differentially expressed miRNAs in sera and exosomes

Based on predicted targets of miRNAs using Targetscan and mirRanda as well as functional analysis using Ingenuity Pathway Analysis (IPA), the functional categories of miRNAomes in sera and exosomes were identified. Further functional analysis of uniquely expressed miRNAs in sera and exosomes revealed 3,847 and 1,520 gene targets, respectively. Among the top 20 functions of predicted gene targets of the uniquely expressed miRNAs in sera and exosomes, 14 functional categories were common in two types of samples (Table 4). Besides the common functions, the sera uniquely expressed miRNAs targeted the function of diseases and disorders such as neurological/infections diseases and skeletal, muscular, hereditary and psychological disorders, while the exosomes uniquely expressed miRNAs packaged into exosomes targeted the function of tissue development and lipid metabolism (Table 4).

**Table 4:** Top 20 functional categories of predicted gene targets by uniquely expressed miRNAs in sera and exosomes

Sera		Exosomes	
Category	B-H *p*-value	Category	B-H *p*-value
Cellular Growth and Proliferation	1.27E-28-1.11E-03	Cellular Growth and Proliferation	2.76E-12-6.05E-02
Cancer	4.54E-24-1.01E-03	Cancer	1.26E-08-6.05E-02
Organismal Injury and Abnormalities	4.54E-24-1.11E-03	Organismal Injury and Abnormalities	1.26E-08-6.05E-02
Cell Death and Survival	5.07E-24-1.01E-03	Cell Morphology	1.67E-08-6.05E-02
Organismal Survival	5.07E-24-2.65E-06	Organismal Survival	3.05E-08-2.39E-02
Cellular Assembly and Organization	7.61E-20-4.99E-04	Cellular Assembly and Organization	6.75E-07-6.05E-02
Cellular Function and Maintenance	7.61E-20-8.07E-04	Cellular Function and Maintenance	6.75E-07-6.05E-02
Cell Morphology	8.22E-19-7.27E-04	Cellular Movement	3.04E-06-6.03E-02
Molecular Transport	2.63E-16-9.67E-04	Cell Death and Survival	3.43E-06-6.03E-02
Cellular Movement	2.28E-15-1.05E-03	Gastrointestinal Disease	6.85E-06-6.03E-02
Cellular Development	4.18E-15-1.09E-03	Cardiovascular System Development and Function	8.40E-06-6.03E-02
Neurological Disease	3.21E-13-1.11E-03	Molecular Transport	9.36E-06-6.04E-02
Gene Expression	3.57E-13-3.95E-04	Cellular Development	9.36E-06-5.83E-02
Cardiovascular System Development and Function	3.57E-13-1.13E-03	Connective Tissue Development and Function	9.36E-06-5.48E-02
Organismal Development	4.85E-13-1.01E-03	Organismal Development	9.36E-06-6.03E-02
Gastrointestinal Disease	1.43E-12-8.91E-04	Nervous System Development and Function	1.82E-05-5.83E-02
Infectious Disease	1.63E-12-6.47E-04	Tissue Development	1.82E-05-5.83E-02
Skeletal and Muscular Disorders	3.74E-12-1.13E-03	Organ Morphology	5.23E-05-6.03E-02
Hereditary Disorder	8.81E-12-8.81E-12	Lipid Metabolism	5.97E-05-6.03E-02
Psychological Disorders	8.81E-12-7.60E-11	Small Molecule Biochemistry	5.97E-05-6.03E-02

There were 3,183 genes putatively targeting by the 12 DE miRNAs. Functional analysis revealed that most of the functional categories were shared by the highly expressed DE miRNAs in sera or exosomes (Table 5). Additionally, the function of infectious disease and cell-mediated immune response were targeted by sera enriched miRNAs, while the skeletal/muscular system development and lipid metabolism were targeted by exosomes enriched miRNAs. Besides, the most targeted pathways predicted based on sera enriched miRNAs were LPS-stimulated MAPK signaling, while the exosomes enriched miRNAs targeted cancer pathways (Table 6).

**Table 5:** Top 20 functions of predicted gene targets of differentially expressed miRNAs in sera and exosomes

Highly expressed miRNAs in sera		Highly expressed miRNAs in exosomes	
Category	B-H *p*-value	Category	B-H *p*-value
Cellular Assembly and Organization	6.09E-06-9.75E-02	Cell Death and Survival	6.84E-14-3.44E-02
Cellular Function and Maintenance	6.09E-06-9.75E-02	Cellular Assembly and Organization	8.85E-14-2.77E-02
Molecular Transport	1.09E-05-9.68E-02	Cellular Function and Maintenance	8.85E-14-3.45E-02
Cancer	6.32E-05-9.68E-02	Cellular Growth and Proliferation	4.07E-13-3.45E-02
Cell Death and Survival	9.31E-05-9.68E-02	Cell Morphology	1.09E-10-3.34E-02
Organismal Survival	9.31E-05-6.44E-02	Cellular Movement	8.05E-09-2.79E-02
Cellular Growth and Proliferation	9.31E-05-9.75E-02	Cancer	2.61E-08-3.24E-02
Cell Morphology	2.20E-04-9.75E-02	Molecular Transport	4.45E-07-3.21E-02
Cardiovascular System Development and Function	2.94E-04-9.68E-02	Cardiovascular System Development and Function	8.40E-07-3.43E-02
Organismal Development	2.94E-04-9.68E-02	Organismal Development	8.40E-07-3.45E-02
Infectious Disease	7.70E-04-9.68E-02	Organismal Survival	1.38E-06-2.44E-02
Gene Expression	7.70E-04-9.68E-02	Tissue Development	1.79E-05-3.34E-02
Cellular Movement	8.00E-04-9.68E-02	Gene Expression	2.31E-05-2.13E-02
Cell-mediated Immune Response	1.23E-03-9.68E-02	Cellular Development	2.89E-05-3.45E-02
Cellular Development	1.23E-03-9.75E-02	Nervous System Development and Function	5.47E-05-3.23E-02
Hematological System Development and Function	1.23E-03-9.75E-02	Skeletal and Muscular System Development and Function	6.43E-05-3.23E-02
Hematopoiesis	1.23E-03-9.75E-02	Neurological Disease	6.97E-05-3.18E-02
Lymphoid Tissue Structure and Development	1.23E-03-9.75E-02	Lipid Metabolism	1.19E-04-3.18E-02
Tissue Development	1.82E-03-9.75E-02	Small Molecule Biochemistry	1.19E-04-3.21E-02
Connective Tissue Development and Function	2.01E-03-9.75E-02	Psychological Disorders	1.55E-04-2.44E-02

**Table 6:** Predicted pathways targeted by miRNAs differentially expressed in sera and exosomes

	*P*	Genes present in pathway (%)	Molecule
Sera			
LPS-stimulated MAPK Signaling	4.11E-06	24.7	18
Molecular Mechanisms of Cancer	1.40E-05	13.7	51
UVC-Induced MAPK Signaling	3.39E-05	28.6	12
IL-17A Signaling in Gastric Cells	4.32E-05	36.0	9
Notch Signaling	6.28E-05	28.9	11
Exosomes			
Molecular Mechanisms of Cancer	2.48E-10	20.1	75
β Cell Receptor Signaling	3.39E-08	23.2	42
Integrin Signaling	3.53E-08	22.4	45
NGF Signaling	3.65E-08	27.4	31
HGF Signaling	4.43E-08	27.8	30

## Discussion

To date, the searching of blood miRNA biomarkers mainly focused on the altered expression levels of miRNAs in diseases. However, little is known about their expression levels associated with the physiological changes and composition of miRNAome, especially in cattle. Here, we conducted a comprehensive miRNAome comparison between cattle sera and exosomes that have been commonly used in miRNA studies.

While surveying the profiles of cell-free small RNAs in cattle, we observed that the annotated miRNAs were low from the sequencing datasets. Size distribution of the total reads revealed two peaks in sera and exosomes, which is similar as reported in the blood samples of mouse and human [33, 34]. The nucleic acid fragments ranging 19–25 nt were mostly annotated as miRNAs. It has been reported that, in addition to miRNAs, several classes of small RNAs mediated multiple biological functions, including siRNAs (20–24 nt), piRNAs (25–32 nt), and others that bound by Argonaute proteins also could be detected in blood [35–37]. Moreover, studies on the processed small RNAs, such as 5’ tRNA halves and and 5’ YRNA fragments (30–33 nt) suggested that they were abundant in circulating small RNAs and might act as novel form of signaling molecules [38, 39]. Thus, it is postulated that the 30–33 nt peak in our study might be processed small RNAs.

The miRNAs detected in exosomes represented about 78 % of total miRNAs expressed in sera, suggesting exosomes are the major carriers for miRNAs in bovine sera. In particular, there were 282 miRNAs expressed in all individuals with high correlation between sera and exosomes, indicating those miRNAs may play important roles in extra-cellular blood fluid of cattle. MiR-486, miR-92a, miR-16, miR-144, and miR-451 have been commonly applied as erythrocyte markers for human and mice [40, 41], and they were all expressed in our bovine sera and exosomes dataset with high proportion. Besides, miR-22-3p, the secondly abundant miRNA in sera (12.0 %) and exosomes (10.4 %), was miRNA biomarker expressed by myeloid [42]. Therefore, red blood cells and myeloid might contribute significantly to circulating miRNAs and this could have important implications for circulating miRNA biomarker studies. Some of top abundance miRNAs detected in bovine sera were not abundant ones in human [17], vice versa, which were not found in bovine sera. The inconsistence in miRNA spectrums might come from the different erythropoiesis between human and bovine [42].

At present, little is known about the origin and destination of these sera/exosomes miRNAs in the circulatory system. Based on identified miRNA profiles, our results showed that spectra of miRNAomes were different between sera and exosomes, and miRNAome of sera was more complex than that of exosomes. The complexity of sera miRNAomes is reasonable, because circulating miRNAs were bounded to various carriers including exosomes, proteins, lipoprotein particles, and cell-derived extracellular vesicles [28, 29, 43]. For example, it has been reported that Argonaute2 complexes carried a population of circulating miRNAs independent of vesicles in human plasma [44]. It is noticeable that the sera miRNAomes detected in this study may also contain the exosome miRNAs. To precisely identify sera specific miRNAs, sera-depletion from exosomes using size-exclusion chromatography in the future studies will provide the detailed contribution and function of the different circulating miRNAs components in sera. In addition, the unique origin may contribute to the miRNAs patterns in sera and exosomes. The assumption above could be supported by the fact that 24 and 3 miRNAs were exclusively expressed with high level in sera and exosomes, respectively. In addition, 12 miRNAs were differentially expressed between two samples. Because of their distinct profiles, miRNAs from sera and exosomes might reflect different aspects of physiological and pathological conditions in cattle.

Many miRNAs are evolutionarily conserved [45]. Therefore, information from human studies on the highly conserved miRNAs that have important roles in regulating health may also be applied to cattle. For example, miR-196, which was uniquely expressed in sera, has recently been reported to be involved in the antiviral immunity in human [46]. And antiviral interferon response could induce the expression of miR-196, which then inhibited the gene expression and replication of hepatitis C virus [47, 48]. Mitogen activated protein kinases (MAPK) are a group of signaling molecules that play important roles in the inflammatory processes [49–51]. Together with LPS-stimulated MAPK pathway that is the most enriched for up-regulated miRNAs in sera, our results suggest that sera uniquely expressed miRNAs (such as miR-196) may play important roles in regulating inflammation and immune response in cattle. MiR-9 is a brain specific miRNA [52], and we found that miR-9-3p and miR-9-5p was uniquely expressed in bovine sera and exosomes, respectively. The differential enrichment of miR-9-3p and miR-9-5p further demonstrated the specific function of sera and exosomes. In addition, miR-92b was differentially expressed between sera and exosomes, with higher expression in exosomes. Nass et al. reported that miR-92b was specifically expressed in the heart and muscle, and overexpression of miR-92b reduced Mef2 levels and caused muscle attachment defects [53]. In addition, miR-92b and miR-9 were over-expressed in developing brain and neuronal stem cells compared to the adult brain, and thus have been implicated as players in human nervous system development [54, 55]. These suggest that exosomes uniquely expressed and/or highly expressed miRNAs may be used as markers to study changes in development.

The high correlation of expression profiles among individuals was observed, demonstrating the low individual variation of miRNAs in bovine extra-cellular blood fluid, the reasonable approach of sera/exosomes sample preparation and miRNA profiling in this study. Overall, the identified expression of miRNAs in sera and exosomes enabled an insight into the basis miRNAs pattern of healthy cattle, which provides a strong basis to inform the further investigation of circulating miRNAomes as biomarkers in cattle.

## Conclusion

Using RNA-seq, our results revealed that 1) exosomes showed a less complex with respect to the miRNAomes as compared to sera; 2) sera and exosomes had their specificities in profiling of miRNAs, which may reflect different aspects of physiological and pathological conditions in cattle; 3) exosomes were major miRNAs carriers in cattle sera, and both samples could be used as potential candidates in biomarker study for different goals in future. Nevertheless, this study laid a foundation for understanding the dysregulations diverging from ordinary physiology by providing information about miRNAomes of healthy individual of bovine sera and exosomes. Furthermore, our results provide the guidance in selection of sample type for particular goals in cattle miRNAs study. Based on our preliminary findings, sera may be preferable for selecting biomarkers of heath such as disease, while exosomes may be better for monitoring the muscle development and lipid metabolism, which should be validated in the future studies using disease and production deficient animal models.

## Methods

### Blood sample collection

The whole blood samples were collected from 4 healthy lactating Holstein cow (33 ± 7 mon, 654 ± 54 kg; mean ± SD). All cows had normal milk production and intake and did not have any incidence of health related issues (data not shown). Experimental protocols were reviewed and approved by the Livestock Animal Care Committee of the University of Alberta and all procedures were conducted following the guidelines of Canadian Council on Animal Care. Briefly, 9 mL whole blood was collected in S-Movovette Sera-Gel tubes (Sarstedt, Nümbrecht, Germany) containing clot activator, and then the samples were incubated at room temperature for 1 h. The whole blood samples were firstly centrifuged at 4 °C (1,900 × g, 10 min), followed by a second centrifugation at 4 °C (16,000 × g, 10 min). After this step, the circulating cell-free nucleic acid were in the supernatant (sera) and then the sera were transferred into a fresh 2.0 mL tube and stored at -80 °C for further analysis.

### Total RNA isolation

Total RNA in sera was extracted using Total RNA Purification kit (Norgen, Thorold, CA) following manufacturer’s protocols. For exosomal RNA extraction, exosomes were firstly isolated from the same sera samples using the Total Exosomes Isolation kit (Invitrogen, Carlsbad, CA), and then the total RNA was extracted using Total Exosome RNA and Protein Isolation Kit (Invitrogen, Carlsbad, CA). The concentration of RNA was determined using the Qubit microRNA assay kit and the 2.0 Fluorometer (Invitrogen, Carlsbad, CA). The RNA samples were stored at -80 °C for further analysis.

### Library construction and sequencing

Total RNA (5 μL) from each sample was used to construct miRNA library using the TruSeq Small RNA Sample Preparation Kit (Illumina, San Diego, CA) according to the manufacturer’s instruction. Small RNA libraries were then pooled together in equal volumes for gel purification. The pooled library was sequenced at Génome Québec (Montréal, Canada) using the Illumina HiSeq 2000 system (Illumina) as 50 bp single reads.

### Small RNA sequence analysis

Low-quality reads were removed from raw data using CASAVA 1.8 based on chastity. High-quality reads were then subjected to sRNAbench for adaptor sequence trimming and reads length distribution analysis [56]. The sequences with read length larger than 15 nt were aligned against bovine miRNA database (miRBase, release version 21) with the default parameters to identify known miRNAs using sRNAbench. Each library was processed separately. The expression level of miRNAs in each library was estimated by sRNAbench, which normalized reads count number of each miRNA RPM by the following formula: RPM = (miRNA reads number/total mapped reads per library) × 1,000,000. Correlations were calculated using the Pearson method in R software (version 3.0). The differentially expressed (DE) miRNAs were investigated by using bioinformatics tool edgeR [57]. The DE miRNAs were determined by Log_2 _fold change (FC) >1 or < -1 and false discovery rate (FDR) < 0.05 based on Benjamini and Hochberg multiple testing correction [58].

### Target gene prediction and functional analysis

The functional analysis of selected miRNAs was performed following a previous study [59]. Briefly, target genes of selected miRNAs were commonly predicted by TargetScan Relase 6.0 (http://www.targetscan.org/) [60] and miRanda (http://www.microrna.org/microrna/home.do). Then the predicted target genes were analyzed through IPA using core analysis module (www.ingenuity.com). A threshold of FDR < 0.05 and molecule number > 1 were used to enrich significant biological functions of each miRNA.

### Experimental validation of miRNA expression using stem-loop RT-qPCR

The expression of uniquely expressed and DE miRNAs that were identified by miRNA-seq were validated by stem-loop RT-qPCR using TAQMAN miRNA assays following the manufacturer’s instruction (Applied Biosystems, Foster City, CA). Briefly, cDNAs were reverse transcribed from 5 μL of total RNA using 5 X specific miRNA RT primer and then were amplified using a 20 X TAQMAN miRNA assay. Fluorescence signal was detected with StepOnePlus™ Real-Time PCR System (Applied Biosystems, Foster City, CA). In this study, miR-93 was used as an internal reference to calculate the relative expression of target miRNA using the 2^-ΔΔCT^ (cycle threshold, CT) method [61]. This miRNA was selected due to its stable expression between sera and exosomes after comparing with the most used reference U6 [62, 63], and previously reported serum miRNA reference candidates miR-127 and miR-744 in bovine and mouse [64, 65]. In addition, no difference was observed between sera and exosomes in miR-93 expression detected by RNA-seq (*P* > 0.05). Differences were considered statistically different at *P* < 0.05 and analysis were performed using *t*-test.

## Additional files

Additional file 1: Table S1. Compilation of evaluated reads data in the process of generating miRNAs. (DOCX 70 kb) Additional file 2: Table S2. Excel spreadsheet containing total miRNAs expressed in sera and exosomes. Expression data for the miRNAs were normalized to RPM, and the miRNAs that were detected in at least 2 animals in each group were considered expressed in this study. (DOCX 206 kb) Additional file 3: Table S3. Pearson correlation of the detected miRNAs among individuals in sera. (DOCX 38 kb) Additional file 4: Table S4. Pearson correlation of the detected miRNAs among individuals in exosomes. (DOCX 38 kb)

## References

[1] Seegers H, Fourichon C, Beaudeau F (2002). Production effects related to mastitis and mastitis economics in dairy cattle herds. Vet Res.

[2] Schwaiger T, Beauchemin KA, Penner GB (2013). The duration of time that beef cattle are fed a high-grain diet affects the recovery from a bout of ruminal acidosis: dry matter intake and ruminal fermentation. J Anim Sci.

[3] Wierenga K, McAllister TA, Gibb DJ, Chaves AV, Okine EK, Beauchemin KA, Oba M (2010). Evaluation of triticale dried distillers grains with solubles as a substitute for barley grain and barley silage in feedlot finishing diets. J Anim Sci.

[4] Vergara CF, Döpfer D, Cook NB, Nordlund KV, McArt JAA, Nydam DV, Oetzel GR (2014). Risk factors for postpartum problems in dairy cows: explanatory and predictive modeling. J Dairy Sci.

[5] Gilad S, Meiri E, Yogev Y, Benjamin S, Lebanony D, Yerushalmi N (2007). Sera microRNAs are promising novel biomarkers. PLoS One.

[6] Wang K, Zhang S, Marzolf B (2009). Circulating microRNAs, potential biomarkers for drug-induced liver injury. Proc Natl Acad Sci U S A.

[7] Qin X, Xu H, Gong W, Deng W (2013). The Tumor Cytosol miRNAs, Fluid miRNAs, and Exosomes miRNAs in Lung Cancer. Front Oncol.

[8] Ambros V (2004). The functions of animal microRNAs. Nature.

[9] Qin X, Yan L, Zhao X, Li C, Fu Y (2012). microRNA-21 overexpression contributes to cell proliferation by targeting PTEN in endometrioid endometrial cancer. Oncol Lett.

[10] Kim YJ, Hwang SH, Cho HH, Shin KK (2012). MicroRNA 21 regulates the proliferation of human adipose tissue derived mesenchymal stem cells and high‐fat diet‐induced obesity alters microRNA 21 expression in white adipose tissues. J Cell Physiol.

[11] Zhang Z, Sun J, Bai Z, Li H, He S, Chen R (2015). MicroRNA-153 acts as a prognostic marker in gastric cancer and its role in cell migration and invasion. Onco Targets Ther.

[12] Burgos KL, Javaherian A, Bomprezzi R, Ghaffari L, Rhodes S, Courtright A (2013). Identification of extracellular miRNA in human cerebrospinal fluid by next-generation sequencing. RNA.

[13] Wu F, Guo NJ, Tian H, Marohn M, Gearhart S, Bayless TM (2011). Peripheral blood microRNAs distinguish active ulcerative colitis and Crohn’s disease. Inflamm Bowel Dis.

[14] Hoekstra M, van der Lans CAC, Halvorsen B, Gullestad L, Kuiper J, Aukrust P (2010). The peripheral blood mononuclear cell microRNA signature of coronary artery disease. Biochem Biophys Res Commun.

[15] Meder B, Keller A, Vogel B, Haas J, Sedaghat-Hanmedani F, Kayvanpour E (2011). MicroRNA signatures in total peripheral blood as novel biomarkers for acute myocardial infarction. Basic Res Cardiol.

[16] Leidinger P, Backes C, Meder B, Meese E, Keller A (2014). The human miRNA repertoire of different blood compounds. BMC Genomics.

[17] Cheng L, Sharples RA, Scicluna BJ, Hill AF (2014). Exosomes provide a protective and enriched source of miRNA for biomarker profiling compared to intracellular and cell-free blood. J Extracell Vesicles.

[18] Zubakov D, Boersma AW, Choi Y, van Kuijk PF, Wiemer EA, Kayser M (2010). MicroRNA markers for forensic body fluid identification obtained from microarray screening and quantitative RT-PCR confirmation. Int Legal Med.

[19] Weber JA, Baxter DH, Zhang S, Huang DY, Huang KH, Lee MJ (2010). The microRNA spectrum in 12 body fluids. Clin Chem.

[20] Tzimagiorgis G, Michailidou EZ, Kritis A, Markopoulos AK, Kouidou S (2011). Recovering circulating extracellular or cell-free RNA from bodily fluids. Cancer Epidemiol.

[21] Mitchell PS, Parkin RK, Kroh EM, Fritz BR, Wyman SK, Pogosova-Agadjanyan EL (2008). Circulating microRNAs as stable blood-based markers for cancer detection. Proc Natl Acad Sci U S A.

[22] Chen X, Ba Y, Ma L, Cai X, Yin Y, Wang K (2008). Characterization of microRNAs in sera: a novel class of biomarkers for diagnosis of cancer and other diseases. Cell Res.

[23] Ioannidis J, Donadeu FX (2016). Circulating miRNA signatures of early pregnancy in cattle. BMC Genomics.

[24] Zheng Y, Chen KL, Zheng XM, Li HX, Wang GL (2014). Identification and bioinformatics analysis of microRNAs associated with stress and immune response in serum of heat-stressed and normal Holstein cows. Cell Stress Chaperones.

[25] Han W, Zhu Y, Su Y, Li G, Qu L, Zhang H (2016). High-throughput sequencing reveals circulating miRNAs as potential biomarkers for measuring puberty onset in chicken (*Gallus gallus*). PLoS One.

[26] Andersson P, Gidlöf O, Braun OO, Götberg M, van der Pals J, Olde B (2012). Plasma levels of liver-specific miR-122 is massively increased in a porcine cardiogenic shock model and attenuated by hypothermia. Shock.

[27] Ashby J, Flack K, Jimenez LA, Duan Y, Khatib A-KK, Somlo G (2014). Distribution profiling of circulating microRNAs in sera. Anal Chem.

[28] Raposo G, Nijman HW, Stoorvogel W, Liejendekker R, Harding CV, Melief CJ (1996). B lymphocytes secrete antigen-presenting vesicles. J Exp Med.

[29] Denzer K, van Eijk M, Kleijmeer MJ, Jakobson E, de Groot C, Geuze HJ (2000). Follicular dendritic cells carry MHC class II-expressing microvesicles at their surface. J Immunol.

[30] Hu G, Drescher KM, Chen XM (2011). Exosomal miRNAs: biological properties and therapeutic potential. Front Genet.

[31] Kourembanas S (2015). Exosomes: vehicles of intercellular signaling, biomarkers, and vectors of cell therapy. Annu Rev Physiol.

[32] Pritchard CC, Cheng HH, Tewari M (2012). MicroRNA profiling: approaches and considerations. Nat Rev Gene.

[33] Dhahbi JM, Spindler SR, Atamna H, Boffelli D, Mote P, Martin DI (2013). 5’-YRNA fragments derived by processing of transcripts from specific YRNA genes and pseudogenes are abundant in human sera and plasma. Physiol Genomics.

[34] Dhahbi JM, Spindler SR, Atamna H, Yamakawa A, Boffelli D, Mote P (2012). 5’ tRNA halves are present as abundant complexes in sera, concentrated in blood cells, and modulated by aging and calorie restriction. BMC Genomics.

[35] Okamura K (2012). Diversity of animal small RNA pathways and their biological utility. Wiley Interdiscip Rev RNA.

[36] Zhang C (2009). Novel functions for small RNA molecules. Curr Opin Mol Ther.

[37] Esteller M (2011). Non-coding RNAs in human disease. Nat Rev Genet.

[38] Kramer BS (2004). The science of early detection. Urol Oncol.

[39] Mestdagh P, Hartmann N, Baeriswyl L, Andreasen D, Bernard N, Chen C (2014). Evaluation of quantitative miRNA expression platforms in the microRNA quality control (miRQC) study. Nat Methods.

[40] Rasmussen KD, Simmini S, Abreu-Goodger C, Bartonicek N, Di Giacomo M, Bilbao-Cortes D (2010). The miR-144/451 locus is required for erythroid homeostasis. J Experi Med.

[41] Pritchard CC, Kroh E, Wood B, Arroyo JD, Dougherty KJ, Miyaji MM (2012). Blood cell origin of circulating microRNAs: A cautionary note for cancer biomarker studies. Cancer Pre Res (Phila).

[42] Spornraft M, Kirchner B, Pfaffl MW, Riedmaier I (2015). Comparison of the miRNAome and piRNome of bovine blood and plasma by small RNA sequencing. Biotechnol Lett.

[43] Vickers KC, Palmisano BT, Shoucri BM, Shamburek RD, Remaley AT (2011). MicroRNAs are transported in plasma and delivered to recipient cells by high-density lipoproteins. Nat Cell Biol.

[44] Arroyo JD, Chevillet JR, Kroh EM, Ruf IK, Pritchard CC, Gibson DF (2011). Argonaute2 complexes carry a population of circulating microRNAs independent of vesicles in human plasma. Proc Natl Acad Sci U S A.

[45] Siomi H, Siomi MC (2010). Posttranscriptional regulation of MicroRNA biogenesis in animals. Mol Cell.

[46] Mahajan VS, Drake A, Chen J (2009). Virus-specific host miRNAs: antiviral defenses or promoters of persistent infection?. Trends Immunol.

[47] Hou W, Tian Q, Zheng J, Bonkovsky HL (2010). MicroRNA‐196 represses Bach1 protein and hepatitis C virus gene expression in human hepatoma cells expressing hepatitis C viral proteins. Hepatology.

[48] Pedersen IM, Cheng G, Wieland S, Volinia S, Croce CM, Chisari FV (2007). Interferon modulation of cellular microRNAs as an antiviral mechanism. Nature.

[49] Ajizian SJ, English BK, Meals EA (1999). Specific inhibitors of p38 and extracellular signal-regulated kinase mitogen-activated protein kinase pathways block inducible nitric oxide synthase and tumor necrosis factor accumulation murine macrophages stimulated with lipopolysaccharide and interferon-gamma. J Infect Dis.

[50] Haddad EB, Birrell M, McCluskie K, Ling A, Webber SE, Foster ML (2001). Role of p38 MAP kinase in LPS-induced airway inflammation in the rat. Br J Pharmacol.

[51] Schmeck B, Zahlten J, Moog K, van Laak V, Huber S, Hocke AC (2004). Streptococcus pneumoniae-induced p38 MAPK-dependent phosphorylation of RelA at the interleukin-8 promotor. J Biol Chem.

[52] Jung HJ, Coffinier C, Choe Y, Beigneux AP, Davies BS, Yang SH (2012). Regulation of prelamin A but not lamin C by miR-9, a brain-specific microRNA. Proc Natl Acad Sci U S A.

[53] Nass D, Rosenwald S, Meiri E, Gilad S, Tabibian-Keissar H, Schlosberg A (2009). MiR-92b and miR‐9/9* are specifically expressed in brain primary tumors and can be used to differentiate primary from metastatic brain tumors. Brain Pathol.

[54] Zhao JJ, Hua YJ, Sun DG, Meng XX, Xiao HS, Ma X (2006). Genome-wide microRNA profiling in human fetal nervous tissues by oligonucleotide microarray. Childs Nervs Syst.

[55] Kapsimali M, Kloosterman WP, de Bruijn E, Rosa F, Plasterk RH, Wilson SW (2007). MicroRNAs show a wide diversity of expression profiles in the developing and mature central nervous system. Genome Biol.

[56] Barturen G, Rueda A, Hamberg M (2014). sRNAbench: profiling of small RNAs and its sequence variants in single or multi-species high-throughput experiments. Meth Next Generation Sequencing.

[57] Robinson MD, McCarthy DJ, Smyth GK (2010). edgeR: a Bioconductor package for differential expression analysis of digital gene expression data. Bioinformatics.

[58] Benjamini Y, Hochberg Y (1995). Controlling the false discovery rate: a practical and powerful approach to multiple testing. J R Statist Soc B.

[59] Liang G, Malmuthuge N, McFadden TB, Bao H, Griebel PJ, Stothard P (2014). Potential regulatory role of microRNAs in the development of bovine gastrointestinal tract during early life. PLoS One.

[60] Enright AJ, John B, Gaul U, Tuschl T, Sander C (2003). MicroRNA targets in Drosophila. Genome Biol.

[61] Livak KJ, Schmittgen TD (2001). Analysis of relative gene expression data using real-time quantitative PCR and the 2(T)(-Delta Delta C) method. Methods.

[62] Ng EK, Chong WW, Jin H, Lam EK, Shin VY, Yu J (2009). Differential expression of microRNAs in plasma of patients with colorectal cancer: a potential marker for colorectal cancer screening. Gut.

[63] Tsujiura M, Ichikawa D, Komatsu S, Shiozaki A, Takeshita H, Konishi H (2010). Circulating microRNAs in plasma of patients with gastric cancers. Br J Cancer.

[64] Mi QS, Weiland M, Qi RQ, Gao XH, Poisson LM, Zhou L (2012). Identification of mouse serum miRNA endogenous references by global gene expression profiles. PLoS One.

[65] Bae IS, Chung KY, Yi J, Kim TI, Choi HS, Cho YM (2015). Identification of reference genes for relative quantification of circulating microRNAs in bovine serum. PLoS One.

