# Supplementary material for: Comparative miRNAome analysis revealed different miRNA expression profiles in bovine sera and exosomes
Source: BMC Genomics. 2016 Aug 12;17:630. doi: 10.1186/s12864-016-2962-1 (PMC4983018; doi:10.1186/s12864-016-2962-1)
Supplement: Additional file 1: Table S1. — Compilation of evaluated reads data in the process of generating miRNAs. (DOCX 70 kb) [file 12864_2016_2962_MOESM1_ESM.docx]

| Data evaluation | Animal 1 | Animal 2 | Animal 3 | Animal 4 | Mean | SD |
| --- | --- | --- | --- | --- | --- | --- |
| Sera |  |  |  |  |  |  |
| Total sequences | 6246059 | 7724303 | 6502779 | 9219554 | 7423174 | 1360194 |
| Passed quality trimming | 6240867 | 7717830 | 6497375 | 9211478 | 7416888 | 1358878 |
| Unpassed quality trimming | 5192 | 6473 | 5404 | 8076 | 6286 | 1318 |
| Passed length trimming (16 to 40 nt) | 4468074 | 5746675 | 4986976 | 7543656 | 5686345 | 1344934 |
| Unpassed length trimming (<16 or > 40 nt) | 1772793 | 1971155 | 1510399 | 1667822 | 1730542 | 193284 |
| Sequences in analysis | 4468074 | 5746675 | 4986976 | 7543656 | 5686345 | 1344934 |
| Exosomes |  |  |  |  |  |  |
| Total sequences | 1408400 | 1765051 | 1951294 | 1457016 | 1645440 | 257905 |
| Passed quality trimming | 1407269 | 1763701 | 1949747 | 1455909 | 1644157 | 257701 |
| Unpassed quality trimming | 1131 | 1350 | 1547 | 1107 | 1284 | 207 |
| Passed length trimming (16 to 40 nt) | 754719 | 1136054 | 1139008 | 930865 | 990162 | 184742 |
| Unpassed length trimming (<16 or > 40 nt) | 652550 | 627647 | 810739 | 525044 | 653995 | 118171 |
| Sequences in analysis | 754719 | 1136054 | 1139008 | 930865 | 990162 | 184742 |

Table S1. Compilation of evaluated reads data in the process of generating miRNAs
