# Supplementary material for: Comparative miRNAome analysis revealed different miRNA expression profiles in bovine sera and exosomes
Source: BMC Genomics. 2016 Aug 12;17:630. doi: 10.1186/s12864-016-2962-1 (PMC4983018; doi:10.1186/s12864-016-2962-1)
Supplement: Additional file 2: Table S2. — Excel spreadsheet containing total miRNAs expressed in sera and exosomes. Expression data for the miRNAs were normalized to RPM, and the miRNAs that were detected in at least 2 animals in each group were considered expressed in this study. (DOCX 206 kb) [file 12864_2016_2962_MOESM2_ESM.docx]

Table S2. Normalized reads of miRNomes in cattle sera and exosomes

|  | Sera | | | | Exosomes | | | |
| --- | --- | --- | --- | --- | --- | --- | --- | --- |
| miRNA ID | Animal1 | Animal2 | Animal3 | Animal4 | Animal1 | Animal2 | Animal3 | Animal4 |
| bta-let-7a-3p | 71 | 61 | 76 | 49 | 66 | 39 | 39 | 89 |
| bta-let-7a-5p | 1755 | 1766 | 2712 | 1723 | 1483 | 1754 | 2685 | 2352 |
| bta-let-7b | 1160 | 1134 | 1783 | 1269 | 363 | 593 | 1074 | 1236 |
| bta-let-7c | 479 | 395 | 521 | 440 | 297 | 273 | 527 | 361 |
| bta-let-7d | 741 | 524 | 1163 | 635 | 692 | 491 | 1162 | 723 |
| bta-let-7e | 157 | 164 | 272 | 179 | 132 | 140 | 107 | 127 |
| bta-let-7f | 3223 | 2626 | 5307 | 2727 | 4120 | 2534 | 4998 | 3532 |
| bta-let-7g | 3099 | 2796 | 3637 | 2501 | 1697 | 1536 | 2480 | 1756 |
| bta-let-7i | 3122 | 3047 | 3656 | 3299 | 2109 | 2316 | 3465 | 3063 |
| bta-miR-100 | 400 | 447 | 332 | 583 | 247 | 288 | 59 | 260 |
| bta-miR-101 | 12306 | 12097 | 10677 | 17562 | 13612 | 9372 | 10406 | 16721 |
| bta-miR-103 | 13695 | 10390 | 11285 | 10880 | 8553 | 5286 | 7536 | 6455 |
| bta-miR-106a | 19 | 14 | 34 | 7 | 0 | 0 | 20 | 19 |
| bta-miR-106b | 1430 | 2079 | 1666 | 1512 | 890 | 897 | 810 | 875 |
| bta-miR-107 | 13104 | 10036 | 10519 | 10344 | 7943 | 5092 | 6941 | 6036 |
| bta-miR-10a | 5760 | 4041 | 5299 | 6823 | 7729 | 3899 | 4783 | 6506 |
| bta-miR-10b | 20331 | 19435 | 19451 | 31329 | 31558 | 19430 | 17493 | 30055 |
| bta-miR-122 | 41 | 108 | 72 | 13 | 0 | 31 | 49 | 19 |
| bta-miR-1224 | 0 | 0 | 4 | 2 | 0 | 0 | 0 | 0 |
| bta-miR-1246 | 1381 | 437 | 895 | 368 | 560 | 257 | 322 | 374 |
| bta-miR-1247-5p | 19 | 7 | 26 | 11 | 132 | 23 | 10 | 63 |
| bta-miR-1249 | 0 | 3 | 19 | 13 | 16 | 39 | 68 | 63 |
| bta-miR-125a | 1643 | 1284 | 884 | 2223 | 1368 | 780 | 498 | 1224 |
| bta-miR-125b | 599 | 388 | 363 | 505 | 330 | 211 | 225 | 260 |
| bta-miR-1260b | 4 | 0 | 8 | 11 | 0 | 23 | 10 | 19 |
| bta-miR-126-3p | 1718 | 1911 | 2466 | 2564 | 1368 | 1053 | 1152 | 1357 |
| bta-miR-126-5p | 8994 | 11343 | 12180 | 15739 | 5916 | 5973 | 7644 | 9644 |
| bta-miR-127 | 404 | 252 | 597 | 482 | 346 | 234 | 342 | 615 |
| bta-miR-1271 | 15 | 12 | 23 | 13 | 0 | 0 | 0 | 0 |
| bta-miR-128 | 3069 | 2559 | 3252 | 2566 | 1813 | 1349 | 1660 | 1458 |
| bta-miR-129=bta-miR-129-5p | 0 | 2 | 0 | 2 | 0 | 0 | 0 | 0 |
| bta-miR-1291 | 7 | 2 | 0 | 0 | 0 | 0 | 0 | 0 |
| bta-miR-1296 | 15 | 14 | 34 | 11 | 16 | 62 | 29 | 25 |
| bta-miR-1306 | 75 | 44 | 87 | 43 | 148 | 327 | 439 | 152 |
| bta-miR-1307 | 341 | 291 | 521 | 271 | 214 | 242 | 605 | 361 |
| bta-miR-130a | 726 | 420 | 559 | 902 | 659 | 288 | 254 | 380 |
| bta-miR-130b | 1029 | 778 | 1454 | 790 | 956 | 585 | 1015 | 507 |
| bta-miR-132 | 97 | 130 | 204 | 121 | 0 | 55 | 78 | 51 |
| bta-miR-133a | 21761 | 171 | 181 | 215 | 25279 | 156 | 195 | 323 |
| bta-miR-133b | 150 | 3 | 8 | 2 | 148 | 0 | 10 | 0 |
| bta-miR-1343-3p | 183 | 110 | 242 | 108 | 33 | 39 | 156 | 63 |
| bta-miR-136 | 34 | 8 | 45 | 43 | 33 | 0 | 10 | 70 |
| bta-miR-138 | 225 | 290 | 245 | 253 | 297 | 273 | 225 | 197 |
| bta-miR-1388-3p | 344 | 163 | 204 | 206 | 198 | 133 | 127 | 76 |
| bta-miR-1388-5p | 112 | 139 | 140 | 119 | 148 | 39 | 68 | 57 |
| bta-miR-139 | 97 | 117 | 166 | 177 | 33 | 70 | 49 | 95 |
| bta-miR-140 | 4121 | 4263 | 7716 | 9149 | 5043 | 3478 | 4656 | 7165 |
| bta-miR-141 | 2081 | 1008 | 963 | 1756 | 1796 | 725 | 722 | 1763 |
| bta-miR-142-3p | 1018 | 815 | 1492 | 747 | 1022 | 483 | 957 | 932 |
| bta-miR-142-5p | 22932 | 22367 | 20308 | 18181 | 30470 | 20078 | 20851 | 18547 |
| bta-miR-143 | 21742 | 41607 | 16135 | 61665 | 16875 | 22822 | 11070 | 40321 |
| bta-miR-144 | 520 | 749 | 706 | 819 | 379 | 265 | 273 | 387 |
| bta-miR-145 | 273 | 234 | 242 | 381 | 165 | 156 | 146 | 203 |
| bta-miR-1468 | 917 | 1174 | 1802 | 2052 | 890 | 1310 | 1640 | 1959 |
| bta-miR-146a | 655 | 222 | 366 | 193 | 346 | 62 | 107 | 95 |
| bta-miR-146b | 157 | 144 | 185 | 110 | 16 | 39 | 20 | 44 |
| bta-miR-147 | 79 | 59 | 72 | 103 | 16 | 55 | 59 | 82 |
| bta-miR-148a | 9065 | 11844 | 15432 | 12033 | 8240 | 8047 | 10377 | 8490 |
| bta-miR-148b | 382 | 547 | 895 | 440 | 264 | 320 | 342 | 342 |
| bta-miR-149-5p | 41 | 27 | 34 | 79 | 33 | 16 | 88 | 152 |
| bta-miR-150 | 8743 | 13042 | 9472 | 9005 | 12063 | 14487 | 8356 | 10824 |
| bta-miR-151-3p | 3413 | 3048 | 4283 | 3317 | 3098 | 2667 | 2655 | 2397 |
| bta-miR-151-5p | 1748 | 1042 | 2183 | 1059 | 1417 | 655 | 1093 | 1110 |
| bta-miR-152 | 588 | 740 | 721 | 628 | 478 | 351 | 420 | 457 |
| bta-miR-153 | 0 | 3 | 11 | 0 | 0 | 0 | 0 | 0 |
| bta-miR-154b | 4 | 0 | 0 | 2 | 0 | 0 | 0 | 0 |
| bta-miR-154c | 60 | 51 | 76 | 137 | 49 | 55 | 88 | 95 |
| bta-miR-155 | 262 | 127 | 234 | 108 | 231 | 117 | 156 | 101 |
| bta-miR-15a | 7014 | 8562 | 7082 | 7417 | 5834 | 5941 | 5584 | 6709 |
| bta-miR-15b | 528 | 707 | 680 | 639 | 577 | 499 | 420 | 583 |
| bta-miR-16a | 5397 | 6281 | 10235 | 5383 | 2043 | 2316 | 2811 | 2562 |
| bta-miR-16b | 16083 | 22967 | 22688 | 19572 | 9772 | 14043 | 13989 | 14260 |
| bta-miR-17-3p | 79 | 95 | 60 | 99 | 82 | 62 | 39 | 63 |
| bta-miR-17-5p | 779 | 830 | 1318 | 684 | 396 | 343 | 654 | 418 |
| bta-miR-181a | 6022 | 5868 | 8128 | 7796 | 6229 | 4569 | 5828 | 6436 |
| bta-miR-181b | 453 | 369 | 861 | 655 | 428 | 288 | 683 | 634 |
| bta-miR-181c | 146 | 85 | 162 | 105 | 99 | 47 | 98 | 82 |
| bta-miR-181d | 22 | 14 | 19 | 34 | 16 | 16 | 10 | 19 |
| bta-miR-182 | 26 | 19 | 38 | 36 | 66 | 8 | 10 | 25 |
| bta-miR-183 | 0 | 0 | 11 | 9 | 0 | 0 | 0 | 0 |
| bta-miR-1839 | 352 | 308 | 306 | 339 | 247 | 335 | 283 | 406 |
| bta-miR-1842 | 60 | 39 | 136 | 56 | 82 | 78 | 10 | 51 |
| bta-miR-185 | 19 | 24 | 4 | 16 | 16 | 8 | 0 | 6 |
| bta-miR-186 | 9773 | 9172 | 11346 | 9147 | 11503 | 8366 | 8493 | 8154 |
| bta-miR-187 | 0 | 3 | 34 | 7 | 0 | 0 | 0 | 0 |
| bta-miR-188 | 52 | 10 | 15 | 20 | 16 | 0 | 0 | 13 |
| bta-miR-18a | 116 | 127 | 166 | 110 | 99 | 86 | 88 | 120 |
| bta-miR-18b | 4 | 22 | 15 | 11 | 0 | 0 | 0 | 0 |
| bta-miR-191 | 27300 | 21309 | 32855 | 18919 | 27191 | 19056 | 26045 | 16220 |
| bta-miR-192 | 57437 | 56112 | 31446 | 47759 | 57216 | 36717 | 22033 | 32978 |
| bta-miR-193a | 52 | 25 | 30 | 34 | 66 | 8 | 10 | 32 |
| bta-miR-193a-3p | 86 | 46 | 34 | 43 | 132 | 31 | 59 | 95 |
| bta-miR-193b | 82 | 93 | 72 | 54 | 99 | 70 | 117 | 25 |
| bta-miR-194 | 359 | 312 | 257 | 220 | 264 | 203 | 137 | 89 |
| bta-miR-195 | 97 | 124 | 128 | 144 | 66 | 39 | 78 | 101 |
| bta-miR-196a | 0 | 0 | 11 | 9 | 0 | 0 | 0 | 0 |
| bta-miR-196b | 19 | 10 | 23 | 13 | 0 | 0 | 0 | 0 |
| bta-miR-197 | 41 | 53 | 53 | 58 | 49 | 203 | 137 | 101 |
| bta-miR-199a-3p | 5060 | 4216 | 4593 | 7373 | 2966 | 2199 | 3231 | 4641 |
| bta-miR-199a-5p | 180 | 102 | 166 | 227 | 198 | 39 | 49 | 197 |
| bta-miR-199b | 255 | 332 | 279 | 368 | 165 | 211 | 156 | 209 |
| bta-miR-199c | 5060 | 4216 | 4593 | 7375 | 2966 | 2199 | 3231 | 4641 |
| bta-miR-19a | 213 | 337 | 344 | 238 | 544 | 335 | 381 | 431 |
| bta-miR-19b | 1011 | 1223 | 1367 | 913 | 1566 | 1302 | 1191 | 1211 |
| bta-miR-200a | 651 | 361 | 555 | 686 | 346 | 203 | 146 | 292 |
| bta-miR-200b | 34 | 19 | 15 | 29 | 16 | 8 | 20 | 25 |
| bta-miR-200c | 15 | 19 | 30 | 22 | 0 | 0 | 20 | 19 |
| bta-miR-204 | 161 | 119 | 521 | 130 | 363 | 125 | 488 | 184 |
| bta-miR-205 | 981 | 664 | 801 | 1469 | 775 | 288 | 410 | 1135 |
| bta-miR-208b | 644 | 10 | 4 | 4 | 643 | 0 | 49 | 6 |
| bta-miR-20a | 468 | 490 | 593 | 330 | 231 | 187 | 478 | 292 |
| bta-miR-20b | 11 | 15 | 8 | 16 | 0 | 8 | 0 | 6 |
| bta-miR-210 | 393 | 324 | 480 | 417 | 148 | 172 | 264 | 349 |
| bta-miR-211 | 0 | 7 | 8 | 7 | 16 | 16 | 0 | 6 |
| bta-miR-21-3p | 281 | 202 | 404 | 240 | 1335 | 546 | 869 | 526 |
| bta-miR-214 | 135 | 122 | 110 | 204 | 99 | 39 | 49 | 95 |
| bta-miR-215 | 19500 | 14865 | 8407 | 9463 | 20978 | 10097 | 6833 | 6594 |
| bta-miR-21-5p | 26215 | 19354 | 27783 | 24228 | 30783 | 18308 | 25352 | 24792 |
| bta-miR-216a | 0 | 2 | 4 | 2 | 0 | 0 | 0 | 0 |
| bta-miR-217 | 0 | 3 | 0 | 2 | 0 | 0 | 0 | 0 |
| bta-miR-218 | 26 | 24 | 83 | 52 | 16 | 8 | 10 | 32 |
| bta-miR-219-5p | 0 | 5 | 4 | 11 | 16 | 0 | 10 | 6 |
| bta-miR-221 | 5319 | 3648 | 6999 | 3885 | 3279 | 2253 | 4754 | 2479 |
| bta-miR-222 | 1059 | 949 | 2006 | 1003 | 511 | 444 | 1240 | 723 |
| bta-miR-223 | 3391 | 3164 | 4551 | 2245 | 2027 | 1528 | 2567 | 1268 |
| bta-miR-22-3p | 136183 | 105054 | 139084 | 100932 | 130928 | 80778 | 111227 | 93564 |
| bta-miR-224 | 90 | 51 | 125 | 182 | 49 | 47 | 78 | 152 |
| bta-miR-22-5p | 808 | 664 | 540 | 480 | 330 | 351 | 273 | 273 |
| bta-miR-2284a | 0 | 2 | 0 | 2 | 0 | 0 | 0 | 0 |
| bta-miR-2284aa | 371 | 310 | 676 | 287 | 511 | 296 | 703 | 355 |
| bta-miR-2284ab | 34 | 49 | 64 | 47 | 16 | 62 | 78 | 108 |
| bta-miR-2284ac | 4 | 3 | 8 | 7 | 0 | 23 | 10 | 6 |
| bta-miR-2284b | 4 | 0 | 4 | 0 | 0 | 0 | 0 | 0 |
| bta-miR-2284d | 7 | 2 | 4 | 0 | 0 | 0 | 0 | 0 |
| bta-miR-2284e | 4 | 3 | 19 | 4 | 33 | 8 | 10 | 6 |
| bta-miR-2284f | 7 | 3 | 23 | 4 | 33 | 8 | 10 | 6 |
| bta-miR-2284h-5p | 0 | 15 | 30 | 22 | 16 | 16 | 49 | 19 |
| bta-miR-2284j | 0 | 5 | 8 | 9 | 16 | 16 | 10 | 0 |
| bta-miR-2284k | 0 | 0 | 0 | 0 | 16 | 0 | 10 | 6 |
| bta-miR-2284l | 0 | 2 | 0 | 4 | 0 | 8 | 0 | 13 |
| bta-miR-2284m | 0 | 2 | 4 | 4 | 0 | 0 | 10 | 6 |
| bta-miR-2284n | 0 | 2 | 8 | 0 | 0 | 0 | 0 | 0 |
| bta-miR-2284o | 0 | 0 | 4 | 4 | 0 | 0 | 0 | 0 |
| bta-miR-2284w | 90 | 95 | 83 | 105 | 16 | 156 | 176 | 82 |
| bta-miR-2284x | 1153 | 1066 | 1341 | 1153 | 1170 | 1138 | 1542 | 1097 |
| bta-miR-2284y | 1153 | 1066 | 1341 | 1153 | 1170 | 1138 | 1542 | 1097 |
| bta-miR-2284z | 371 | 310 | 676 | 287 | 511 | 296 | 703 | 355 |
| bta-miR-2285aa | 22 | 3 | 15 | 18 | 16 | 0 | 20 | 6 |
| bta-miR-2285ac | 4 | 2 | 8 | 2 | 0 | 8 | 10 | 0 |
| bta-miR-2285b | 7 | 15 | 15 | 11 | 0 | 0 | 0 | 0 |
| bta-miR-2285g | 11 | 24 | 23 | 22 | 33 | 39 | 0 | 19 |
| bta-miR-2285k | 90 | 54 | 151 | 70 | 181 | 70 | 59 | 19 |
| bta-miR-2285l | 4 | 2 | 8 | 4 | 0 | 0 | 0 | 0 |
| bta-miR-2285o | 7 | 0 | 8 | 2 | 0 | 0 | 0 | 0 |
| bta-miR-2285p | 4 | 3 | 4 | 0 | 0 | 0 | 20 | 6 |
| bta-miR-2285q | 22 | 29 | 34 | 18 | 33 | 16 | 59 | 13 |
| bta-miR-2285r | 7 | 2 | 0 | 0 | 0 | 0 | 0 | 0 |
| bta-miR-2285t | 79 | 85 | 117 | 74 | 33 | 8 | 59 | 101 |
| bta-miR-2285u | 7 | 2 | 4 | 2 | 0 | 0 | 0 | 0 |
| bta-miR-2285y | 135 | 76 | 140 | 72 | 132 | 78 | 156 | 127 |
| bta-miR-2285z | 0 | 2 | 0 | 2 | 0 | 16 | 20 | 0 |
| bta-miR-2299-3p | 7 | 2 | 19 | 0 | 0 | 0 | 0 | 0 |
| bta-miR-2299-5p | 4 | 3 | 8 | 0 | 16 | 0 | 0 | 25 |
| bta-miR-2313-5p | 0 | 2 | 0 | 11 | 0 | 0 | 0 | 0 |
| bta-miR-2316 | 4 | 0 | 19 | 4 | 0 | 8 | 29 | 6 |
| bta-miR-2320-5p | 7 | 20 | 19 | 13 | 0 | 16 | 0 | 6 |
| bta-miR-2332 | 0 | 0 | 0 | 0 | 0 | 8 | 0 | 6 |
| bta-miR-2336 | 11 | 2 | 4 | 11 | 0 | 23 | 0 | 6 |
| bta-miR-2339 | 0 | 5 | 0 | 2 | 0 | 0 | 0 | 0 |
| bta-miR-2346 | 0 | 3 | 4 | 2 | 0 | 0 | 0 | 0 |
| bta-miR-2349 | 0 | 2 | 4 | 2 | 0 | 0 | 0 | 0 |
| bta-miR-2368-3p | 0 | 0 | 4 | 4 | 0 | 0 | 0 | 0 |
| bta-miR-2376 | 22 | 14 | 23 | 16 | 0 | 16 | 78 | 19 |
| bta-miR-2388-3p | 7 | 0 | 4 | 2 | 0 | 0 | 0 | 0 |
| bta-miR-2388-5p | 0 | 2 | 19 | 0 | 0 | 0 | 10 | 6 |
| bta-miR-2397-3p | 0 | 5 | 4 | 0 | 0 | 0 | 0 | 0 |
| bta-miR-2397-5p | 0 | 3 | 0 | 4 | 0 | 0 | 0 | 0 |
| bta-miR-2399-5p | 4 | 2 | 8 | 7 | 0 | 0 | 0 | 0 |
| bta-miR-23a | 5962 | 4084 | 5684 | 5872 | 3395 | 1692 | 2870 | 3177 |
| bta-miR-23b-3p | 4057 | 2681 | 3626 | 3891 | 1780 | 756 | 1777 | 1928 |
| bta-miR-24 | 0 | 7 | 0 | 2 | 0 | 0 | 0 | 0 |
| bta-miR-2400 | 0 | 5 | 0 | 9 | 0 | 0 | 0 | 0 |
| bta-miR-2403 | 0 | 27 | 57 | 31 | 0 | 47 | 0 | 25 |
| bta-miR-2409 | 7 | 3 | 0 | 0 | 0 | 0 | 0 | 0 |
| bta-miR-2411-3p | 0 | 3 | 0 | 2 | 0 | 0 | 0 | 0 |
| bta-miR-2415-3p | 34 | 83 | 23 | 67 | 165 | 156 | 39 | 63 |
| bta-miR-2419-5p | 217 | 242 | 1107 | 175 | 247 | 234 | 1201 | 159 |
| bta-miR-2422 | 11 | 2 | 0 | 0 | 0 | 0 | 0 | 0 |
| bta-miR-2425-5p | 4 | 0 | 8 | 4 | 0 | 0 | 0 | 0 |
| bta-miR-2431-3p | 0 | 3 | 0 | 4 | 0 | 0 | 0 | 0 |
| bta-miR-24-3p | 1527 | 1167 | 2988 | 1106 | 280 | 226 | 469 | 336 |
| bta-miR-2443 | 0 | 0 | 4 | 7 | 0 | 0 | 0 | 0 |
| bta-miR-2447 | 0 | 7 | 19 | 0 | 0 | 0 | 0 | 0 |
| bta-miR-2448-3p | 4 | 8 | 11 | 4 | 0 | 0 | 0 | 0 |
| bta-miR-2448-5p | 7 | 2 | 0 | 0 | 0 | 0 | 0 | 0 |
| bta-miR-2453 | 0 | 2 | 4 | 2 | 0 | 0 | 0 | 0 |
| bta-miR-2454-3p | 22 | 22 | 60 | 20 | 0 | 31 | 59 | 0 |
| bta-miR-2457 | 4 | 24 | 19 | 25 | 33 | 70 | 29 | 38 |
| bta-miR-2474 | 0 | 5 | 0 | 4 | 0 | 0 | 10 | 32 |
| bta-miR-2478 | 19 | 14 | 23 | 25 | 16 | 0 | 59 | 32 |
| bta-miR-2483-3p | 7 | 8 | 0 | 11 | 16 | 0 | 10 | 25 |
| bta-miR-2483-5p | 4 | 5 | 0 | 7 | 16 | 0 | 0 | 6 |
| bta-miR-2484 | 0 | 2 | 0 | 7 | 0 | 0 | 0 | 0 |
| bta-miR-25 | 14578 | 21870 | 15799 | 17598 | 12376 | 18783 | 14711 | 15180 |
| bta-miR-26a | 7658 | 5414 | 8626 | 6027 | 6757 | 3774 | 5359 | 5650 |
| bta-miR-26b | 2654 | 2025 | 3456 | 1960 | 1285 | 1014 | 1337 | 1072 |
| bta-miR-26c | 6295 | 4316 | 6995 | 4858 | 5537 | 3189 | 4364 | 4641 |
| bta-miR-27a-3p | 15682 | 10936 | 14133 | 15819 | 10893 | 5115 | 7683 | 9873 |
| bta-miR-27b | 39947 | 36221 | 42275 | 46243 | 28806 | 20701 | 30281 | 31729 |
| bta-miR-28 | 378 | 313 | 412 | 260 | 396 | 242 | 254 | 266 |
| bta-miR-2887 | 19 | 5 | 8 | 0 | 16 | 8 | 10 | 19 |
| bta-miR-2889 | 15 | 10 | 8 | 11 | 0 | 78 | 29 | 44 |
| bta-miR-2890 | 4 | 8 | 4 | 2 | 0 | 8 | 0 | 6 |
| bta-miR-2892 | 4 | 5 | 11 | 4 | 16 | 0 | 10 | 13 |
| bta-miR-2898 | 7 | 7 | 15 | 2 | 33 | 23 | 29 | 0 |
| bta-miR-2903 | 4 | 2 | 4 | 7 | 0 | 0 | 10 | 13 |
| bta-miR-2904 | 22 | 7 | 30 | 16 | 49 | 8 | 10 | 25 |
| bta-miR-2957 | 576 | 729 | 714 | 615 | 428 | 343 | 420 | 457 |
| bta-miR-296-3p | 517 | 513 | 986 | 626 | 264 | 257 | 605 | 501 |
| bta-miR-296-5p | 19 | 15 | 23 | 13 | 33 | 62 | 68 | 70 |
| bta-miR-29a | 6572 | 5075 | 4838 | 4428 | 4664 | 2729 | 3007 | 2999 |
| bta-miR-29b | 112 | 86 | 136 | 83 | 82 | 23 | 78 | 25 |
| bta-miR-29c | 1557 | 1127 | 1409 | 1059 | 1219 | 686 | 918 | 640 |
| bta-miR-29d-3p | 34 | 17 | 19 | 9 | 82 | 8 | 20 | 6 |
| bta-miR-29d-5p | 4 | 8 | 11 | 0 | 0 | 0 | 0 | 0 |
| bta-miR-301a | 1239 | 1264 | 1481 | 1090 | 1088 | 1170 | 1347 | 1078 |
| bta-miR-301b | 292 | 154 | 295 | 119 | 132 | 187 | 449 | 95 |
| bta-miR-30a-5p | 5603 | 7700 | 5027 | 6895 | 6707 | 5294 | 3436 | 4895 |
| bta-miR-30b-5p | 2279 | 1876 | 3244 | 1330 | 1961 | 1060 | 1328 | 1129 |
| bta-miR-30c | 1531 | 1184 | 2610 | 1207 | 2175 | 1458 | 1513 | 1807 |
| bta-miR-30d | 15933 | 10822 | 19923 | 11304 | 18951 | 11321 | 18538 | 11229 |
| bta-miR-30e-5p | 17397 | 15181 | 20240 | 13550 | 17600 | 12312 | 16585 | 12028 |
| bta-miR-30f | 71 | 85 | 64 | 65 | 66 | 101 | 49 | 76 |
| bta-miR-3120 | 4 | 3 | 8 | 18 | 0 | 8 | 0 | 6 |
| bta-miR-32 | 704 | 649 | 842 | 621 | 412 | 335 | 254 | 292 |
| bta-miR-320a | 3148 | 2228 | 3078 | 2647 | 1631 | 1481 | 1523 | 1224 |
| bta-miR-323 | 0 | 5 | 0 | 11 | 0 | 0 | 0 | 0 |
| bta-miR-324 | 30 | 20 | 53 | 20 | 66 | 0 | 29 | 19 |
| bta-miR-326 | 120 | 75 | 91 | 67 | 198 | 62 | 98 | 76 |
| bta-miR-328 | 64 | 81 | 136 | 74 | 494 | 515 | 752 | 457 |
| bta-miR-330 | 153 | 95 | 242 | 65 | 132 | 70 | 59 | 57 |
| bta-miR-331-3p | 7 | 14 | 19 | 20 | 0 | 31 | 39 | 32 |
| bta-miR-331-5p | 71 | 136 | 140 | 123 | 132 | 109 | 88 | 38 |
| bta-miR-335 | 56 | 81 | 72 | 119 | 165 | 47 | 10 | 44 |
| bta-miR-338 | 45 | 31 | 68 | 70 | 66 | 39 | 20 | 57 |
| bta-miR-339a | 378 | 313 | 400 | 296 | 1434 | 1450 | 1259 | 1465 |
| bta-miR-339b | 378 | 313 | 400 | 296 | 1434 | 1450 | 1259 | 1465 |
| bta-miR-33a | 97 | 44 | 91 | 52 | 0 | 78 | 88 | 57 |
| bta-miR-33b | 86 | 66 | 60 | 90 | 132 | 47 | 49 | 63 |
| bta-miR-340 | 4 | 3 | 0 | 7 | 0 | 0 | 0 | 0 |
| bta-miR-342 | 1785 | 1530 | 1473 | 1512 | 2060 | 1084 | 879 | 989 |
| bta-miR-3431 | 75 | 24 | 64 | 130 | 33 | 16 | 49 | 51 |
| bta-miR-345-3p | 34 | 66 | 94 | 58 | 99 | 8 | 78 | 89 |
| bta-miR-345-5p | 7 | 14 | 4 | 2 | 0 | 8 | 10 | 25 |
| bta-miR-34a | 135 | 51 | 208 | 49 | 0 | 16 | 59 | 19 |
| bta-miR-34c | 4 | 10 | 11 | 11 | 16 | 0 | 0 | 6 |
| bta-miR-3596 | 797 | 817 | 1163 | 875 | 214 | 405 | 742 | 875 |
| bta-miR-3604 | 4256 | 3618 | 3913 | 6309 | 2422 | 1887 | 2567 | 3976 |
| bta-miR-361 | 1265 | 934 | 1609 | 1063 | 527 | 374 | 576 | 501 |
| bta-miR-362-3p | 22 | 10 | 19 | 27 | 16 | 0 | 10 | 44 |
| bta-miR-363 | 19 | 42 | 42 | 58 | 16 | 8 | 20 | 51 |
| bta-miR-365-3p | 45 | 54 | 19 | 40 | 99 | 117 | 59 | 51 |
| bta-miR-369-3p | 52 | 25 | 87 | 94 | 49 | 0 | 29 | 19 |
| bta-miR-369-5p | 0 | 2 | 15 | 9 | 0 | 0 | 0 | 0 |
| bta-miR-370 | 0 | 7 | 0 | 9 | 0 | 0 | 0 | 0 |
| bta-miR-371 | 4 | 0 | 11 | 2 | 0 | 0 | 0 | 0 |
| bta-miR-374a | 7 | 36 | 26 | 13 | 33 | 0 | 10 | 19 |
| bta-miR-374b | 60 | 29 | 64 | 63 | 66 | 16 | 88 | 25 |
| bta-miR-375 | 2317 | 2940 | 2497 | 2449 | 1978 | 2643 | 2743 | 2270 |
| bta-miR-376a | 0 | 2 | 4 | 0 | 0 | 0 | 0 | 0 |
| bta-miR-376b | 4 | 3 | 11 | 2 | 0 | 0 | 0 | 0 |
| bta-miR-376d | 0 | 2 | 4 | 0 | 0 | 0 | 0 | 0 |
| bta-miR-378 | 8238 | 2798 | 2855 | 3104 | 7877 | 2292 | 1943 | 2454 |
| bta-miR-378b | 15 | 17 | 11 | 4 | 0 | 8 | 10 | 32 |
| bta-miR-378c | 868 | 398 | 514 | 507 | 972 | 491 | 332 | 457 |
| bta-miR-378d | 56 | 24 | 38 | 52 | 99 | 16 | 29 | 6 |
| bta-miR-380-3p | 45 | 69 | 136 | 148 | 16 | 23 | 39 | 89 |
| bta-miR-381 | 135 | 31 | 60 | 54 | 115 | 23 | 20 | 63 |
| bta-miR-409a | 0 | 2 | 0 | 2 | 0 | 0 | 0 | 0 |
| bta-miR-409b | 15 | 8 | 15 | 20 | 0 | 8 | 0 | 6 |
| bta-miR-410 | 45 | 34 | 45 | 85 | 82 | 39 | 68 | 82 |
| bta-miR-411a | 138 | 85 | 68 | 94 | 99 | 86 | 49 | 152 |
| bta-miR-411c-5p | 0 | 0 | 11 | 7 | 0 | 0 | 0 | 0 |
| bta-miR-421 | 1404 | 918 | 2024 | 1090 | 1071 | 678 | 1308 | 697 |
| bta-miR-423-3p | 11547 | 9897 | 13049 | 9169 | 11486 | 10932 | 11919 | 10354 |
| bta-miR-423-5p | 36332 | 29849 | 41738 | 30555 | 28641 | 27906 | 42289 | 28888 |
| bta-miR-424-3p | 0 | 5 | 8 | 16 | 0 | 0 | 0 | 0 |
| bta-miR-424-5p | 0 | 12 | 23 | 36 | 16 | 0 | 0 | 13 |
| bta-miR-425-3p | 124 | 93 | 381 | 81 | 16 | 8 | 117 | 51 |
| bta-miR-425-5p | 2276 | 1996 | 3097 | 1817 | 2324 | 2019 | 1904 | 1775 |
| bta-miR-429 | 124 | 88 | 91 | 251 | 99 | 62 | 78 | 120 |
| bta-miR-432 | 22 | 25 | 23 | 34 | 0 | 8 | 20 | 19 |
| bta-miR-433 | 0 | 0 | 4 | 9 | 0 | 0 | 10 | 6 |
| bta-miR-449a | 4 | 0 | 0 | 4 | 0 | 0 | 0 | 0 |
| bta-miR-450a | 7 | 7 | 4 | 9 | 16 | 0 | 20 | 0 |
| bta-miR-450b | 19 | 41 | 72 | 101 | 82 | 31 | 29 | 95 |
| bta-miR-451 | 20013 | 38198 | 24278 | 26339 | 21407 | 35742 | 22247 | 28996 |
| bta-miR-452 | 15 | 5 | 11 | 58 | 0 | 31 | 10 | 6 |
| bta-miR-454 | 19 | 5 | 15 | 9 | 16 | 8 | 10 | 25 |
| bta-miR-455-3p | 4 | 2 | 26 | 16 | 16 | 0 | 10 | 6 |
| bta-miR-455-5p | 79 | 78 | 242 | 271 | 66 | 39 | 107 | 114 |
| bta-miR-484 | 674 | 839 | 767 | 666 | 1170 | 1240 | 830 | 786 |
| bta-miR-485 | 0 | 2 | 11 | 7 | 0 | 16 | 10 | 19 |
| bta-miR-486 | 138114 | 200043 | 129743 | 167309 | 173610 | 348852 | 268765 | 279006 |
| bta-miR-487b | 11 | 24 | 15 | 22 | 0 | 0 | 29 | 19 |
| bta-miR-490 | 19 | 2 | 0 | 2 | 0 | 16 | 0 | 6 |
| bta-miR-491 | 0 | 3 | 0 | 2 | 0 | 0 | 0 | 0 |
| bta-miR-493 | 4 | 8 | 26 | 27 | 0 | 0 | 0 | 0 |
| bta-miR-494 | 49 | 19 | 68 | 47 | 16 | 0 | 10 | 6 |
| bta-miR-495 | 7 | 2 | 11 | 0 | 0 | 0 | 0 | 0 |
| bta-miR-497 | 505 | 513 | 567 | 994 | 494 | 405 | 469 | 774 |
| bta-miR-499 | 539 | 19 | 23 | 22 | 231 | 8 | 10 | 13 |
| bta-miR-502a | 15 | 5 | 26 | 9 | 33 | 0 | 10 | 19 |
| bta-miR-502b | 4 | 7 | 4 | 2 | 0 | 8 | 10 | 0 |
| bta-miR-503-3p | 0 | 0 | 8 | 2 | 0 | 0 | 0 | 0 |
| bta-miR-504 | 15 | 22 | 19 | 11 | 33 | 23 | 10 | 6 |
| bta-miR-505 | 120 | 188 | 162 | 168 | 115 | 86 | 137 | 101 |
| bta-miR-532 | 427 | 312 | 415 | 318 | 330 | 257 | 303 | 292 |
| bta-miR-543 | 7 | 2 | 11 | 18 | 0 | 0 | 0 | 0 |
| bta-miR-545-3p | 7 | 3 | 11 | 0 | 0 | 0 | 0 | 0 |
| bta-miR-545-5p | 7 | 15 | 23 | 16 | 16 | 16 | 10 | 13 |
| bta-miR-592 | 0 | 5 | 11 | 11 | 0 | 0 | 0 | 0 |
| bta-miR-599 | 4 | 0 | 4 | 2 | 0 | 0 | 0 | 0 |
| bta-miR-6119-3p | 4 | 8 | 15 | 11 | 33 | 16 | 29 | 6 |
| bta-miR-6119-5p | 389 | 234 | 400 | 247 | 264 | 327 | 390 | 273 |
| bta-miR-6120-3p | 45 | 17 | 19 | 11 | 0 | 16 | 10 | 6 |
| bta-miR-6123 | 26 | 32 | 38 | 38 | 49 | 31 | 49 | 25 |
| bta-miR-628 | 11 | 12 | 11 | 18 | 0 | 0 | 0 | 0 |
| bta-miR-6517 | 4 | 15 | 26 | 13 | 33 | 39 | 20 | 6 |
| bta-miR-6518 | 0 | 2 | 0 | 4 | 0 | 0 | 0 | 0 |
| bta-miR-652 | 344 | 130 | 257 | 182 | 214 | 133 | 215 | 203 |
| bta-miR-6520 | 4 | 3 | 4 | 7 | 0 | 0 | 0 | 0 |
| bta-miR-6523a | 11 | 12 | 15 | 9 | 0 | 0 | 39 | 6 |
| bta-miR-6524 | 30 | 71 | 19 | 63 | 66 | 62 | 49 | 38 |
| bta-miR-6529a | 2062 | 1896 | 3165 | 1660 | 1763 | 1489 | 3553 | 1427 |
| bta-miR-6533 | 0 | 0 | 0 | 0 | 16 | 0 | 0 | 13 |
| bta-miR-654 | 0 | 2 | 0 | 2 | 0 | 0 | 0 | 0 |
| bta-miR-655 | 0 | 0 | 8 | 4 | 0 | 0 | 0 | 0 |
| bta-miR-660 | 2167 | 1889 | 2225 | 2299 | 3461 | 1676 | 1982 | 2530 |
| bta-miR-664b | 0 | 7 | 4 | 9 | 16 | 8 | 20 | 6 |
| bta-miR-665 | 22 | 8 | 38 | 40 | 16 | 31 | 10 | 13 |
| bta-miR-669 | 805 | 669 | 891 | 1092 | 791 | 920 | 996 | 1034 |
| bta-miR-671 | 0 | 3 | 4 | 9 | 0 | 0 | 0 | 0 |
| bta-miR-677 | 22 | 20 | 19 | 54 | 49 | 8 | 68 | 63 |
| bta-miR-708 | 22 | 10 | 8 | 13 | 0 | 0 | 0 | 0 |
| bta-miR-744 | 404 | 229 | 593 | 269 | 148 | 250 | 556 | 393 |
| bta-miR-760-3p | 71 | 59 | 125 | 58 | 16 | 23 | 107 | 25 |
| bta-miR-769 | 318 | 219 | 291 | 184 | 412 | 273 | 469 | 457 |
| bta-miR-7857 | 19 | 17 | 87 | 11 | 16 | 8 | 68 | 6 |
| bta-miR-7859 | 0 | 2 | 0 | 4 | 0 | 0 | 0 | 0 |
| bta-miR-7861 | 4 | 8 | 23 | 9 | 0 | 0 | 0 | 0 |
| bta-miR-874 | 116 | 46 | 121 | 58 | 115 | 39 | 98 | 101 |
| bta-miR-877 | 352 | 290 | 510 | 271 | 247 | 179 | 303 | 197 |
| bta-miR-885 | 7 | 27 | 19 | 0 | 16 | 16 | 10 | 6 |
| bta-miR-92a | 48447 | 57000 | 55290 | 51738 | 61550 | 89613 | 98088 | 74402 |
| bta-miR-92b | 1186 | 981 | 1171 | 1218 | 1945 | 2191 | 2275 | 2042 |
| bta-miR-93 | 3769 | 5942 | 4906 | 4551 | 3032 | 4164 | 3885 | 3189 |
| bta-miR-935 | 4 | 3 | 4 | 0 | 0 | 0 | 0 | 0 |
| bta-miR-9-3p | 15 | 2 | 0 | 4 | 0 | 0 | 0 | 0 |
| bta-miR-95 | 4 | 10 | 19 | 7 | 0 | 0 | 0 | 0 |
| bta-miR-9-5p | 0 | 0 | 0 | 0 | 16 | 0 | 0 | 13 |
| bta-miR-98 | 240 | 268 | 472 | 213 | 313 | 257 | 332 | 279 |
| bta-miR-99a-3p | 37 | 36 | 34 | 29 | 33 | 0 | 29 | 38 |
| bta-miR-99a-5p | 445 | 342 | 313 | 525 | 461 | 140 | 68 | 171 |
| bta-miR-99b | 382 | 336 | 351 | 543 | 396 | 234 | 303 | 457 |

Reads are normalized to reads per million (RPM).
