# Supplementary material for: Comparative miRNAome analysis revealed different miRNA expression profiles in bovine sera and exosomes
Source: BMC Genomics. 2016 Aug 12;17:630. doi: 10.1186/s12864-016-2962-1 (PMC4983018; doi:10.1186/s12864-016-2962-1)
Supplement: Additional file 3: Table S3. — Pearson correlation of the detected miRNAs among individuals in sera. (DOCX 38 kb) [file 12864_2016_2962_MOESM3_ESM.docx]

Table S3. Pearson correlation of the detected miRNAs among individuals in serum

|  | SE1 | SE2 | SE3 | SE4 |
| --- | --- | --- | --- | --- |
| SE1 | 1.00 | 0.95 | 0.98 | 0.95 |
| SE2 | 0.95 | 1.00 | 0.94 | 0.99 |
| SE4 | 0.98 | 0.94 | 1.00 | 0.94 |
| SE4 | 0.95 | 0.99 | 0.94 | 1.00 |
