# Supplementary material for: Comparative miRNAome analysis revealed different miRNA expression profiles in bovine sera and exosomes
Source: BMC Genomics. 2016 Aug 12;17:630. doi: 10.1186/s12864-016-2962-1 (PMC4983018; doi:10.1186/s12864-016-2962-1)
Supplement: Additional file 4: Table S4. — Pearson correlation of the detected miRNAs among individuals in exosomes. (DOCX 38 kb) [file 12864_2016_2962_MOESM4_ESM.docx]

Table S4. Pearson correlation of the detected miRNAs among individuals in exosomes

|  | EX1 | EX2 | EX3 | EX4 |
| --- | --- | --- | --- | --- |
| EX1 | 1.00 | 0.89 | 0.94 | 0.93 |
| EX2 | 0.89 | 1.00 | 0.98 | 0.99 |
| EX3 | 0.94 | 0.98 | 1.00 | 0.99 |
| EX4 | 0.93 | 0.99 | 0.99 | 1.00 |
